# Acute exacerbations of fibrosing interstitial lung disease associated with connective tissue diseases: a population-based study

**DOI:** 10.1186/s12890-019-0960-1

**Published:** 2019-11-14

**Authors:** Mengshu Cao, Jian Sheng, Xiaohua Qiu, Dandan Wang, Dongmei Wang, Yang Wang, Yonglong Xiao, Hourong Cai

**Affiliations:** 1 Deprtment of Respiratory and Critical Care Medicine, Nanjing Drum Tower Hospital, The Affiliated Hospital of Nanjing University Medical School, 321 Zhongshan Road, Nanjing, 210008 Jiangsu China; 2 Department of Rheumatology and Immunology, Nanjing Drum Tower Hospital, The Affiliated Hospital of Nanjing University Medical School, 321 Zhongshan Road, Nanjing, 210008 Jiangsu China; 3 Department of Radiology, Nanjing Drum Tower Hospital, The Affiliated Hospital of Nanjing University Medical School, 321 Zhongshan Road, Nanjing, 210008 Jiangsu China

**Keywords:** Interstitial lung disease, Connective tissue disease, Idiopathic pulmonary fibrosis, Acute exacerbation

## Abstract

**Background:**

Acute exacerbation (AE) is the major cause of morbidity and mortality in patients with idiopathic pulmonary fibrosis (IPF). AEs also occur in other forms of fibrosing interstitial lung disease (fILD). The clinical features and prognosis of AE patients with connective tissue diseases (CTDs) associated-ILD has not been fully described.

**Methods:**

We retrospectively reviewed 177 patients with either IPF or a characterized CTD-ILD admitted to Nanjing Drum Tower Hospital with an AE from January 2010 to December 2016.

**Results:**

The study cohort included 107 subjects with AE-IPF and 70 cases with AE-CTD-ILD. Female gender, prior use of corticosteroid and immunosupressants, lower serum albumin, higher D-dimer level, TLC% pred, survival, and treatment with immunosupressants and caspofungin were more common in the CTD-ILD group (all p<0.05). The incidences of AE-CTD-ILD and AE-IPF were similar in our single center (*p* = 0.526). TLC% pred was the risk factor for AE after ILD diagnosis for 1 year in CTD patients (*p* = 0.018). Log-rank tests showed patients with CTD-ILD had a significantly lower mortality rate compared with IPF patients after AEs (*p* = 0.029). No significant difference in survival was noted among CTD subgroups (*p* = 0.353). The survival was negatively correlated with WBC count, LDH and CT score, (*p* = 0.006, *p* = 0.013 and *p* = 0.035, respectively), and positively correlated with PaO_2_/FiO_2_ ratio (p<0.001) in the CTD-ILD group. WBC count and PO_2_/FiO_2_ ratio were the independent predictors for survival in AE-CTD-ILD after adjusting for other clinical variates in Cox regression Models (*p* = 0.038 and *p* < 0.001, respectively).

**Conclusions:**

The clinical characteristics of patients with AE-CTD-ILD differed from those with AE-IPF, while AE incidences were similar between the two groups. Subjects with AE-CTD-fILD tended to have a better prognosis, and WBC count and PO_2_/FiO_2_ ratio were the independent survival predictors for these patients.

## Background

Idiopathic pulmonary fibrosis (IPF) is a chronic and progressive scarring disease of the lung characterized by worsening dyspnea and lung function over time [1, 2]. Some patients will experience an acute, clinically significant respiratory deterioration characterized by widespread alveolar abnormality [3]. Acute exacerbations (AEs) are a major cause of mortality of patients with IPF. The incidence of AE-IPF ranges from 4 to 20% per year among IPF patients in reported clinical trials [4–8], and up to one-half of IPF patients die from AE [9]. In most cases, in-hospital mortality rate was up to 50% [7]. The etiology of AE-IPF is still unknown. Recent evidence suggests that causes of acute lung injury (ALI) and histopathologic diffuse alveolar damage (DAD) such as infection, aspiration, surgery and air pollution exposure could contribute to the development of AE-IPF [3, 10, 11]. Low forced vital capacity (FVC), diffusing capacity for carbonmonoxide (DLCO) and baseline oxygenation were known risk factors for AE [7, 10, 12–14]. Lower baseline FVC and DLCO and more extensive CT abnormalities at the time of AE and worse oxygenation were predictors of mortality [7, 8, 15]. The elevated levels of Kerbs von Lungen-6 and leptin in peripheral blood could be the risk and prognostic factors for AE-IPF [16, 17].

Recently, AE has also been shown to occur in fibrosing interstitial lung diseases (fILD) other than IPF, such as connective tissue disease (CTD) associated ILD, idiopathic nonspecific interstitial lung disease and chronic hypersensitivity pneumonias [18–22]. Park et al. reported AE in CTD-ILD was similar to AE-IPF and most common in older patients with rheumatoid arthritis (RA) [18]. Suda et al. suggested that AE occurred mostly with RA-ILD with a usual interstitial pneumonia (UIP) pattern [19]. Toyoda et al. showed some AE cases in patients with CTD had good response to corticosteroid [23]. To date, there are few reports of AE in CTD-ILD patients. The incidence, clinical characteristics and prognosis of AE patients in CTD-ILD have yet to be fully elucidated.

We conducted a retrospective study of AE in CTD-ILD and IPF in our single center. The aims were to clarify the occurrence, clinical characteristics and prognosis of patients with CTD-ILD who developed AE, and to identify risk and predictive factors in Chinese population.

## Methods

### Study subjects

**Fig. 1:**
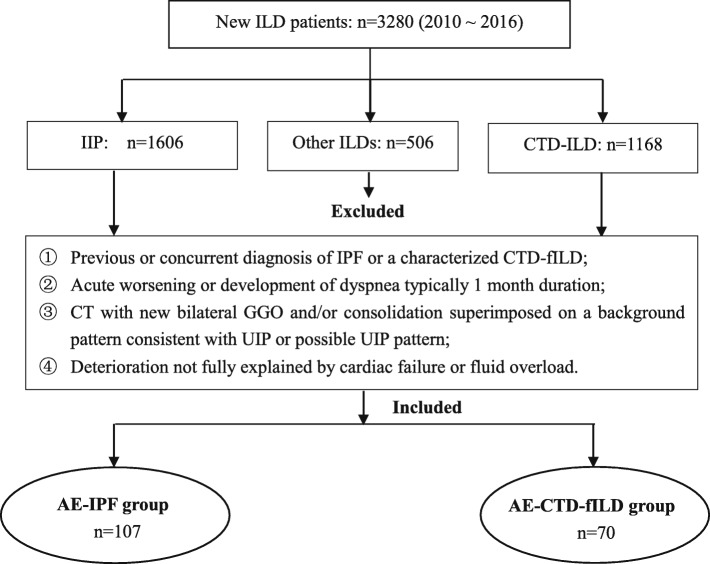
Flow chart of included patients

This retrospective cohort study was approved by the Ethics Committee of Nanjing Drum Tower Hospital and conducted in compliance with the Helsinki Declaration (1989) (NO.2016–160-01). We retrospectively reviewed 3280 new patients with ILD admitted to our hospital from January 2010 to December 2016, and 177 consecutive patients with an AE of underlying fILD were enrolled in our study (Fig. 1). The follow-up period was from 6 months to 7 years. The final cohort included 107 cases of AE-IPF and 70 cases of AE-CTD-ILD. All subjects had an underlying UIP or possible UIP pattern on chest high resolution computed tomography (HRCT) as defined by the American Thoracic Society (ATS)/European Respiratory Society (ERS) guidelines [1, 2]. The diagnosis of the underlying CTD and interstitial pneumonia with autoimmune features (IPAF) was according to the established criteria [23–30]. IPAF means patients with ILD that had features of autoimmunity, yet falling short of a characterizable CTD, was included in CTD-ILD. The definition of AE was based on the updated international criteria for AE-IPF [3]. Briefly, AE-IPF and AE-CTD-fILD were defined as an acute, clinically significant respiratory deterioration characterized by evidence of new widespread alveolar abnormality with: ① Previous or concurrent diagnosis of IPF or a characterized CTD-fILD; ② Acute worsening or development of dyspnea typically 1 month duration; ③ CT with new bilateral ground-glass opacity and/or consolidation superimposed on a background pattern consistent with UIP or possible UIP pattern; ④ Deterioration not fully explained by cardiac failure or fluid overload [3]. All baseline and follow-up clinical data and chest imaging findings were obtained from hospital medical records. Vital status was obtained from medical records or telephone interview. Baseline clinical variables were obtained at the time of AE. The pulmonary function tests (PFTs) were conducted up to 1 month prior or after the initial diagnosis as AE-PF.

### HRCT scanning

Chest HRCT examination was performed within 24 h of the diagnosis of respiratory failure with 1.0–1.5 mm thick sections with appropriate window settings (window width: 1600, window level: − 600). The findings were blindly reviewed and scored by two senior radiologists without any knowledge of clinical data. The images were assessed for the presence and extent of ground glass opacity, consolidation, traction bronchiectasis, reticulation, honeycombing and emphysema. The overall extent of abnormalities was determined for per lung using a 4-point scale (0 = no involvement, 1 = 1–25% involvement, 2 = 26–50% involvement, 3 = 51–75% involvement, and 4 = 76–100% involvement) according to the published studies [31, 32]. The HRCT of each case was evaluated as UIP pattern and possible UIP (P-UIP) pattern. Those with an inconsistent with UIP pattern were excluded from this study [1, 33].

### Statistical analysis

Data are presented as mean ± standard deviation (SD) for continuous variables or percentages for categorical variables. Chi-squared and Fisher’s exact tests were used for categorical data, and the unpaired t-test and Kruskal-Wallis test were used for continuous data. A multivariate Cox regression model was used to identify significant variables capable of predicting AE or acting as prognostic factors. Bivariate correlation analysis was used to identify the relationship between total survival time and clinical variables. The incidence of AE was obtained from the Kaplan-Meier (K-M) survival curve by treating AE as the death variable. Survival was also evaluated using the log rank test. *P* < 0.05 was considered to represent statistical significance. Statistical analyses were performed by using IBM SPSS version 19 (SPSS, Inc., Chicago IL, USA) and Prism version 5 (GraphPad, SanDiego, CA, USA).

## Results

### Clinical characteristics

The baseline clinical features of subjects with AE-CTD-fILD (*n* = 70) and AE-IPF (*n* = 107) were summarized in Table 1. The average ages were similar. Female gender, prior use of corticosteroid and immunosupressants were more common in the CTD-ILD group (p<0.001, *p* = 0.011 and p<0.001, respectively). Serum albumin (ALB), D-dimer and TLC% pred also differed between the two groups (p<0.001, *p* = 0.023 and *p* = 0.012, respectively). Subjects with AE-CTD-ILD were treated with immunosupressants and caspofungin more frequently than AE-IPF subjects after AEs (p<0.001 and *p* = 0.009, respectively).

**Table 1:** Baseline clinical features

	n	All patients	AE-IPF group	AE-CTD-ILD group	*p* value
Clinical Characteristics					
Gender (M/F)	177	116/61	81/26	35/35	< 0.001
Age (years old)	177	67.46 ± 9.72	68.52 ± 9.87	65.84 ± 9.33	0.073
Smoking history (Y/N)	177	56/121	38/69	18/52	0.170
Prior corticosteroids use (Y/N)	177	125/52	68/39	57/13	0.011
Prior immunosuppressant use (Y/N)	177	39/138	3/104	36/34	< 0.001
Prior NAC use (Y/N)	177	153/24	91/16	62/8	0.503
Reduction or Discontinuation of corticosteroids before AE (Y/N)	177	57/120	34/73	23/47	0.880
Fever (Y/N)	177	105/72	62/43	43/29	0.861
WBC count (×10^9^)	177	11.12 ± 4.55	11.26 ± 4.91	10.91 ± 3.97	0.619
ESR (mm/h)	177	39 (0–120)	39 (0–120)	40 (0–101)	0.275
CRP (mg/L)	177	50.2 (0.2–246)	47 (0.2–243.5))	56.05 (0.2–246)	0.603
LDH (U/L)	177	439.26 ± 174.13	438.28 ± 158.32	440.76 ± 197.06	0.927
ALB (g/L)	177	32.14 ± 3.48	32.88 ± 3.45	31.01 ± 3.23	< 0.001
BNP (pg/ml)	125	94.30 (0–1130)	104 (5–828)	88.15 (0–1130)	0.869
PaO2/FiO2 ratio	177	126 (34–357)	121 (45–246)	140.50 (34–357)	0.050
D-dimer (mg/L)	153	1.84 (0.02–24.82)	1.46 (0.02–22.4)	2.06 (0.10–24.82)	0.023
CT Score	177	8 (4–8)	8 (4–8)	8 (4–8)	0.772
FVC (L)	105	2.02 (0.74–4.61)	2.05 (0.74–3.36)	1.86 (0.86–4.61)	0.886
FVC % pred	105	63.22 ± 18.39	60.33 ± 17.32	66.18 ± 19.20	0.170
TLC% pred	89	53.15 ± 12.81	48.80 ± 13.53	57.67 ± 10.45	0.012
DLCO % pred	89	41.66 ± 15.59	38.66 ± 14.78	44.78 ± 16.09	0.163
PAH (mmHg)	89	42 ± 9.848	41.97 ± 9.642	42.03 ± 10.222	0.980
BMI (kg/m^2^)	105	24.44 (17.58–34.11)	24.44 (17.99–30.53)	24.52 (17.58–34.11)	0.836
Anti-fibrotic therapy (Y/N)	3	3/176	3/104	0/70	0.279
Treatments after AE					
Mechanical Ventilation (MV, Y/N)	177	93/84	57/50	36/34	0.810
Maximal dosage of methylprednisonle (mg/d)	177	240 (40–1000)	240 (40–1000)	240 (40–1000)	0.252
Immunosuppressant (Y/N)	177	58/119	14/93	44/26	< 0.001
Immunoglobulin (Y/N)	177	72/105	43/64	29/41	0.869
Co-trimoxazole (Y/N)	177	99/78	57/50	42/28	0.378
Caspofungin (Y/N)	177	38/139	16/91	22/48	0.009

AE = acute exacerbation; IPF = idiopathic pulmonary fibrosis; fILD = fibrosing interstitial lung disease; CTDs = connective tissue diseases; NAC = N-acetylcysteine; WBC = white blood cell; ESR = erythrocyte sedimentation rate; CRP = C reactive protein; LDH = lactate dehydrogenase; ALB = albumin; BNP = B-type natriuretic peptide; PaO2/FiO2 = Oxygenation index; CT = Computed tomography; UIP = usual interstitial pneumonia; P-UIP = possible UIP; FVC = forced vital capacity; TLC = total lung capacity; DLCO = diffusing capacity of the lung for CO2; PAH = pulmonary arterial hypertension; BMI = body mass index; MV = mechanical ventilation

### AE occurrence in patients with CTD-ILD

**Fig. 2:**
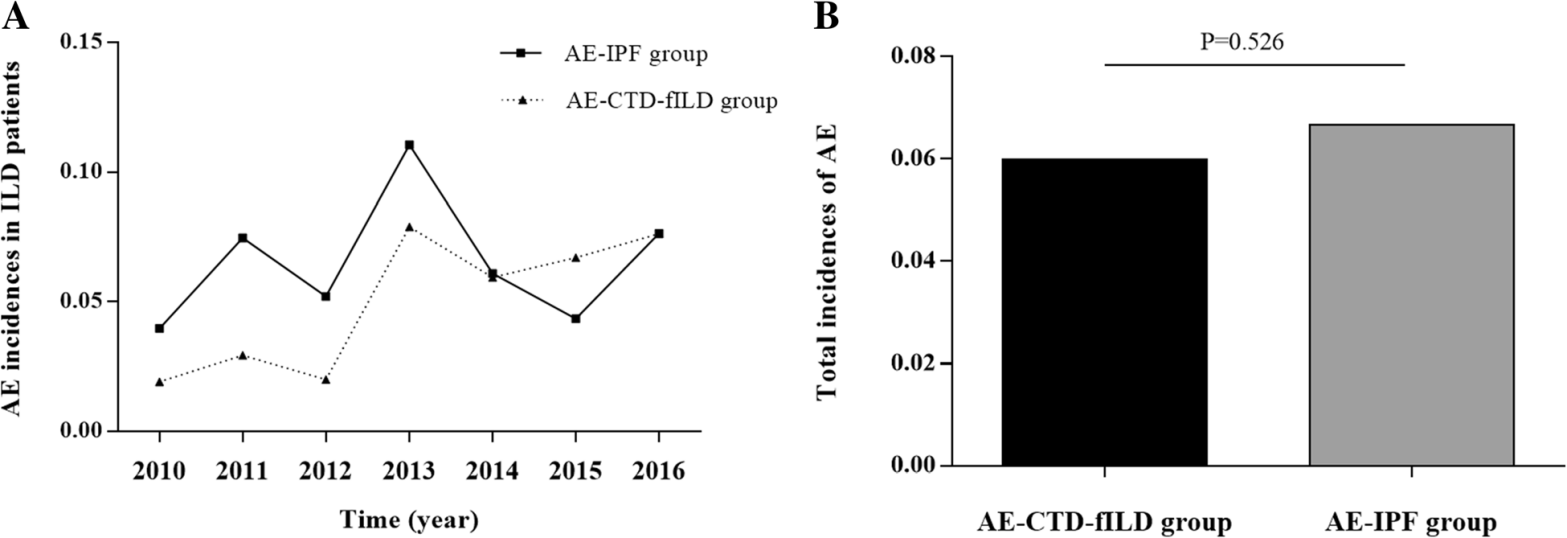
AE occurrence in patients with CTD-ILD **a** The occurrence of AE-IPF in IIP and AE-CTD-fILD in CTD-ILD from 2010 to 2016 in our center.**b**: Comparison of AE incidence between the two groups

The incidence of AE-IPF in patients with idiopathic interstitial pneumonia (IIP) was 3.97–11.06% (6.67%) and the incidence of AE-CTD-fILD in CTD-ILD cases was 1.92–7.89% (5.99%) per year in our single center from January 2010 to December 2016 (Fig. 2A). The difference of AE occurrence between the two groups was not significant (*p* = 0.526) (Fig. 2B).

In univariate Cox analysis, prior corticosteroids use was the independent risk factor for AE among in subjects with CTDs after ILD diagnosis for 1 year (HR: 0.415, 95% CI: 0.215–802, *p* = 0.009), and pulmonary arterial hypertension (PAH) was the independent risk factor for AE occurrence in IPF patients (HR: 0.959, 95% CI: 0.921–1.000, *p* = 0.048) (Table 2). After considering the clinical significance and adjusting other clinical variables, prior corticosteroids and immunosuppressant use, FVC, TLC% pred, PAH and body mass index (BMI) were included in the multivariate Cox analysis. The findings showed only TLC% pred was associated with the occurrences of AE among CTD-ILD patients (HR: 0.668, 95% CI: 0.505–0.938; p:0.018). However, none of above clinical variables could predict the occurrence of AE in AE-IPF group (Table 2).

**Table 2:** Risk factors for AE occurrence after ILD diagnosis for 1 year by univariate and multivariate Cox regression models in the two groups

Clinical Variables	Univariate Cox Model			Multivariate Cox Model		
	HR	95.0%CI	p value	HR	95.0% CI	p value
AE-IPF group						
Gender	0.950	0.578–1.559	0.838	–	–	–
Age (years old)	1.009	0.986–1.032	0.441	–	–	–
Smoking history	1.347	0.858–2.113	0.195	0.499	0.103–2.407	0.386
Prior corticosteroids use	0.802	0.511–1.258	0.336	0.654	0.087–4.909	0.680
Prior immunosuppressant use	0.638	0.157–2.599	0.531	–	–	–
Corticosteroids reduction or discontinuation	0.818	0.516–1.298	0.393	–	–	–
FVC (L)	0.719	0.415–1.245	0.239	0.284	0.024–3.393	0.320
TLC% pred	1.015	0.983–1.048	0.371	1.279	0.957–1.709	0.096
DLCO% pred	0.984	0.958–1.011	0.244	–	–	–
PAH (mmHg)	0.959	0.921–1.000	0.048	0.950	0.875–1.031	0.217
BMI (kg/m^2^)	0.932	0.815–1.066	0.304	0.743	0.534–1.032	0.077
AE-CTD-fILD group						
Gender	0.751	0.434–1.301	0.308	–	–	–
Age (years old)	0.995	0.963–1.028	0.765	–	–	–
Smoking history	0.654	0.335–1.277	0.214	2.567	0.076–86.493	0.599
Prior corticosteroids use	0.415	0.215–0.802	0.009	0.049	0.002–1.487	0.083
Prrior immunosuppressant use	0.727	0.421–1.253	0.251	–	–	–
Corticosteroids reduction or discontinuation	0.702	0.385–1.280	0.248	–	–	–
FVC (L)	0.857	0.559–1.313	0.478	3.521	0.306–40.477	0.312
TLC% pred	0.964	0.921–1.009	0.117	0.688	0.505–0.938	0.018
DLCO% pred	1.000	0.969–1.033	0.986	–	–	–
PAH (mmHg)	1.026	0.986–1.068	0.209	1.351	0.974–1.873	0.072
BMI (kg/m^2^)	0.935	0.856–1.022	0.137	0.823	0.666–1.016	0.823

AE = acute exacerbation; IPF = idiopathic pulmonary fibrosis; fILD = fibrosing interstitial lung disease; CTDs = connective tissue diseases; HR = hazard ratio; 95% CI = 95% confidence interval; TLC = total lung capacity; DLCO = diffusing capacity of the lung for CO2; PAH = pulmonary arterial hypertension; BMI = body mass index

### Survival

**Fig. 3:**
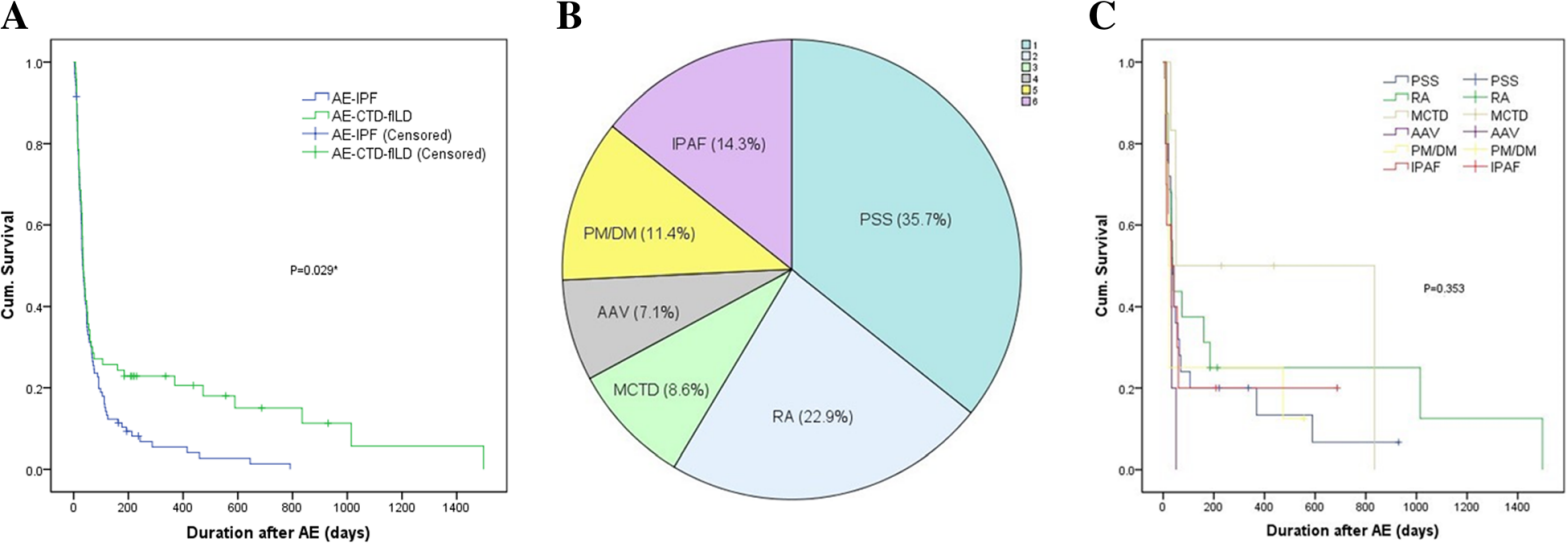
The survival of patients with AE-CTD-fILD **a** Comparison of the survival between patients with AE-CTD-ILD and AE-IPF by Kaplan-Meier method. **b**: The percentages of patients in different CTD subgroups. **c**: Comparison of the survival in different CTD-ILD subgroups by Kaplan-Meier method

The median survival time of AE-CTD-ILD patients was 35.0 ± 4.2 days. Kaplan-Merier method analysis revealed that AE-CTD-ILD subjects had a significantly better overall survival than AE-IPF patients (log-rank test, *p* = 0.029) (Fig. 3A). The differences of cumulative proportion survival (CPS) less than 180 days after AE were not significant between the two groups (Additional file 1: Table S1). Among 70 cases of AE-CTD-ILD, 25 (35.7%) were primary Sjogren syndrome (PSS), 16 (22.9%) were RA, 6 (8.6%) were mixed connective tissue disease (MCTD), 5 (7.1%) were antineutrophil cytoplasmic antibodyassociated vasculitis (AAV), 8 (11.4%) were PM/DM and 10 (14.3%) were IPAF (Fig. 3B). There was no difference in survival among different CTDs subgroups (log-rank, *p* = 0.353) (Fig. 3C).

### Prognostic factors

Bivariate correlation analysis demonstrated that overall survival time was negatively correlated with WBC counts, LDH, and CT score (r = − 0.323, *p* = 0.006; r = − 0.296, *p* = 0.013 and r = − 0.252, *p* = 0.035), respectively, and positively correlated to PaO_2_/FiO_2_ (r = 0.407, < 0.001) in AE-CTD-ILD group. In AE-IPF group, the findings were similar to CTD-ILD patients except for a negative correlation with the maximal dosage of corticosteroid and no correlation with PaO_2_/FiO_2_ (Table 3).

**Table 3:** Bivariate correlation analysis between clinical variables and overall survival time

Clinical Variables	AE-IPF group		AE-CTD-fILD group	
	r	p value	r	p value
WBC count (× 10^9^)	−0.277	0.004	−0.323	0.006
LDH (U/L)	−0.220	0.023	−0.296	0.013
PaO2/FiO2 ratio	0.158	0.104	0.407	< 0.001
CT score	−0.433	< 0.001	−0.252	0.035
Maximal dosage of methylprednisolone (mg/d)	−0.196	0.043	−0.146	0.227

WBC: white blood cell; LDH: lactate dehydrogenase; PaO2/FiO2 ratio: Oxygenation index; HRCT: high resolution computed tomography; MV: mechanical ventilation

The univariate analysis showed that WBC count, CRP, LDH, ALB, D-dimmer, PaO_2_/FiO_2_, CT score, treatment with MV, maximal dosage of methylprednisolone, immunosuppressant, immunoglobulin, co-trimoxazole and caspofungin for AE were associated with survival in all 177 cases (all p<0.05, respectively), while only WBC count, PaO_2_/FiO_2_ and CT score were the independent risk factors for survival in these patients (HR 1.051, 95%CI 1.013–1.090, *p* = 0.001; HR 0.991, 95%CI 0.987–0.994, p<0.001 and HR 1.337, 95%CI 1.063–1.682, p<0.013, respectively) (Table 4). As shown in Table 5, similar to all 177 patients, WBC count, PaO_2_/FiO_2_ ratio and CT score in AE-IPF group were also the independent risk factors for survival by a multivariate Cox regression analysis (HR 1.067, 95%CI 1.028–1.108, p = 0.001; HR 0.993, 95%CI 0.989–0.998, *p* = 0.002 and HR 1.531, 95%CI 1.170–2.002, p = 0.002, respectively).

**Table 4:** Prognostic factors for survival by univariate and multivariate Cox regression models in all patients

Clinical Variables	Univariate Cox Model			Multivariate Cox Model		
	HR	95.0% CI	p value	HR	95.0% CI	p value
WBC count (×10^9^)	1.075	1.041–1.109	<0.001	1.051	1.013–1.090	0.008
CRP (mg/L)	1.003	1.000–1.006	0.038	1.003	0.999–1.007	0.157
LDH (U/L)	1.003	1.002–1.004	<0.001	1.000	0.998–1.001	0.430
ALB (U/L)	0.943	0.987–0.992	0.022	1.000	0.999–1.001	0.545
D-dimmer	1.048	1.018–1.080	0.002	1.021	0.971–1.074	0.421
PaO2/FiO2 ratio	0.991	0.998–0.994	<0.001	0.991	0.987–0.994	<0.001
CT score	1.402	1.195–1.645	<0.001	1.337	1.063–1.682	0.013
MV	2.877	2.070–4.000	<0.001	–	–	–
Maximal dosage of methylprednisolone (mg/d)	1.001	1.000–1.001	0.016	1.000	0.999–1.001	0.840
Immunosuppressant	0.673	0.478–0.947	0.023	–	–	–
Immunoglobulin	2.103	1.524–2.901	<0.001	–	–	–
Co-trimoxazole	1.382	1.009–1.892	0.044	–	–	–
Caspofungin	2.057	1.406–3.009	<0.001	–	–	–

WBC: white blood cell; LDH: lactate dehydrogenase; PaO2/FiO2ratio: Oxygenation index; MV: mechanical ventilation; HRCT: high resolution computed tomography

**Table 5:** Prognostic factors for survival by C by univariate and multivariate Cox regression models in the two groups

Clinical Variables	Univariate Cox Model			Multivariate Cox Model		
	HR	95.0% CI	p value	HR	95.0% CI	p value
AE-IPF group						
WBC count (×10^9^)	1.071	1.033–1.111	< 0.001	1.069	1.031–1.109	< 0.001
LDH (U/L)	1.002	1.001–1.003	0.003	–	–	–
PaO2/FiO2 ratio	0.993	0.989–0.997	0.001	0.993	0.989–0.997	0.002
CT score	1.703	1.343–2.158	< 0.001	1.573	1.211–2.042	0.001
MV	2.673	1.747–4.087	< 0.001	–	–	–
Maximal dosage of methylprednisolone (mg/d)	1.001	1.000–1.002	0.006	1.000	1.000–1.001	0.534
Immunoglobulin	2.182	1.427–3.338	< 0.001	–	–	–
Caspofungin	1.809	1.038–3.153	0.036	–	–	–
AE-CTD-fILD group						
WBC count (×10^9^)	1.098	1.027–1.175	0.006	1.074	1.004–1.150	0.038
LDH (U/L)	1.004	1.002–1.005	< 0.001			
PaO2/FiO2 ratio	0.989	0.985–0.994	< 0.001	0.989	0.984–0.994	< 0.001
CT score	1.253	0.993–1.582	0.058	0.975	0.746–1.274	0.852
MV	3.495	2.020–6.047	< 0.001			
Maximal dosage of methylprednisolone (mg/d)	1.000	0.999–1.001	0.613	0.999	0.998–1.000	0.277
Immunoglobulin	2.370	1.401–4.009	0.001	–	–	–
Caspofungin	2.741	1.563–4.808	< 0.001	–	–	–

WBC: white blood cell; LDH: lactate dehydrogenase; PaO2/FiO2 ratio: Oxygenation index; MV: mechanical ventilation; HRCT: high resolution computed tomography

In AE-CTD-ILD group, WBC counts, LDH, PaO_2_/FiO_2_ ratio, treatment with MV, immunoglobulin, and caspofungin for AE were associated with the survival by univariate Cox model (all p<0.05, respectively). After considering the clinical significance and adjusting other clinical variables, WBC count, PaO_2_/FiO_2_, CT score and maximal dosage of methylprednisolone were included in multivariate Cox model. The findings showed that WBC count and PaO_2_/FiO_2_ ratio were the independent prognostic factors for survival by a multivariate Cox regression analysis (HR 1.074, 95%CI 1.004–1.150, *p* = 0.038 and HR 0.989, 95%CI 0.984–0.994, p<0.001, respectively) (Table 5).

## Discussion

The present study retrospectively compared the clinical, chest imaging and follow-up data between 70 subjects with AE-CTD-ILD and 107 subjects with AE-IPF. The clinical features of patients with AE-CTD-ILD differed from those of AE-IPF. Subjects with AE-CTD-ILD had a better survival than AE-IPF patients, while the survival of AE cases in the CTD subgroups didn’t show any difference. WBC count and PO_2_/FiO_2_ ratio could predict the survival of AE-CTD-ILD patients independently.

Recently, the definition and diagnostic criteria of AE-IPF have been updated. IPF patients manifesting an acute, clinically significant respiratory deterioration characterized by evidence of new widespread alveolar abnormality on chest imaging or histopathology should be considered as having an AE [3]. Data showed that a potential infectious aetiology is up to one-third of patients with ILD and acute respiratory decline [22]. The updated criteria of AE-IPF do not exclude respiratory infection [3]. The causes of acute lung injure (ALI) including infection, aspiration, drugs and surgery may lead to events that are indistinguishable from idiopathic AE of IPF [3]. These events are further classified into “triggered” (ie, postprocedure, drug toxicity, infection, aspiration) or “idiopathic” (ie, no inciting event identified) AEs in the updated guideline [3].

The published reports about AE in patients with CTD-ILD have been relatively small series and show inconsistent results. The incidence of AE in RA-UIP was about 11.1–20% [18, 19]. The mild elevation of mean PAH was very common in CTD-ILD patients [34]. Suda et al. suggested that the clinical characteristics of AE in CTD-ILD were similar to those of IPF with high mortality [19]. Park and Suda reported that AE occurred mostly with RA-UIP with poor outcome [18, 19]. Song and Huie suggested that CTD-UIP group was younger, included more women and nonsmokers, and showed better survival than the IPF-UIP group [7, 22]. The current study was based on the updated criteria of Collard about AE-IPF [3] and comprised a 177 cases of AE patients with IPF and CTD-ILD. The findings showed that the clinical features, treatment and prognosis of patients with AE-CTD-ILD differed from those with AE-IPF and published reports [7, 8, 18, 35].

The incidence of AE in IPF patients has been reported in a number of studies. Due to different definition and diagnostic criteria of AE, AE incidence in IPF varied greatly, estimated to be in the range of 4–20% per year [4–8]. In patients with CTD-ILD, Suda et al. revealed an overall AE incidence of 7.2% and a 1-year incidence of 1.25% [19]. Compared with AE-IPF, the incidence of AE in CTD-ILD seemed to be lower [19]. In our single center, AE incidence was about 5% in all new ILD patients about 6% per year in those with CTD-ILD. AE incidence of fILD patients in our study is similar to the published reports of IPF population in other countries [4–8]. IPF patients with physiologically and functionally advanced disease are thought to be at higher risk of AE [3]. However, little is known about the causative factors of AE in patients with CTD-ILD. In CTD-ILD patients, age and SS overlapped with PM/DM were the independent risk factors for AE [19, 36]. In our study, AE occurrence in CTD-ILD patients within 1 year after ILD diagnosis was weakly associated with TLC% pred, likely due to the limited sample size. A larger sample prospective study of AE-CTD-ILD is therefore needed to clarify this complication.

The poor survival of patients with AE-IPF is a great challenge. The MST of these patients was about 3–4 months [6, 7]. Nearly half of patients with AE-IPF died in the hospital, and patients with both definite AE-IPF and suspected AE-IPF had a similar clinical outcome [6, 7, 22]. The published reports on AE-CTD-ILD differ from AE-IPF and include a relatively small number of cases. Tachikawa et al. concluded that the survival of AE-CTD-ILD was better than that of advanced IPF (90-day mortality at 33% vs. 69%) [37]. Huie et al. suggested that IPF patients had a lower rate of survival as compared with patients with non-IPF fibrotic disease after acute respiratory decline [22]. Suda et al. suggested that AE incidence in CTD-ILD was similar to that of IPF with poor prognosis [19]. In our cohort study, the overall survival was significant better in subjects with AE-CTD-ILD than AE-IPF cases, despite their similar short-term survival. Usui Y.et al. reported that systemic inflammatory response (SIRS) was the most significant predictor for in-hospital mortality in patients with AE of chronic fILD including IPF and non-IPF [38]. Infection at AE was thought to determine the short-term survival in these patients. In current study, the inflammatory indicators, WBC counts, CRP, LDH and CT scores didn’t show any difference between the two groups, implying that infection was similar between the two groups. So, the similar short-term survival may be associated with the similar infection in the two groups [38]. The better overall survival in CTD-ILD patients may be associated with the favorable response to corticosteroids treatment [23]. Yoshimura et al. also suggested that the diagnosis of IPF might predict a favorable prognosis in patients with chronic fILD [39], thereby a better survival in AE-CTD-fILD. The lack of difference in overall survival among different CTD subgroups may be due to the small sample size in our study.

A number of factors including lower baseline FVC and DLCO, extensive CT abnormalities and CT pattern, worse oxygenation, higher bronchoalveolar lavage neutrophil and lymphocyte percentages at the time of AE were reported to be associated with the survival of patients with AE-IPF [7, 8, 15, 32, 40, 41]. Literature also indicated that described age, TLC, honeycombing score and higher initial MPAP were the independent prognostic factors in AE-CTD-ILD [34, 35]. In current study, WBC count and PO_2_/FiO_2_ ratio could predict the survival of patients with AE-CTD-ILD after adjusting for other clinical variables. Now, infection has been regarded as one of the causes for AE in IPF patients [3]. WBC is a non-specific inflammation marker of infection. Infection with elevated WBC counts was correlated with the poor clinical outcomes in AE-CTD-fILD patients in our study. Low PO_2_/FiO_2_ ratio could account for the severity of hypoxia and clinical condition at AE, which indicated patients with more severe condition when AE occurred would have worse prognosis. The different risk factors of prognosis in the two groups may be due to the underlying diseases and the conditions at AE occurrence.

Although there is no effective therapy for AE-IPF, supportive care including supplemental oxygen and palliation of symptoms are regarded as important treatment modality. The use of MV is controversial [1, 3]. Corticosteroids are recommended but with somewhat low-quality evidence [1, 3]. There is no consensus on the dosage and course of therapy. A slight survival benefit was reported for the treatment of AE-IPF combining cyclosporine with systemic glucocorticoids as compared to that without cyclosporine in a small retrospective study [41]. The therapy of steroids combined with cyclophosphosphamide for patients with AE-CTD-ILD appeared better than those without any immunosuppressant [7, 42]. However, there is no evidence supporting the benefit of a specific immunosuppressant. Addition of intravenous thrombomodulin and polymyxin B immobilized fiber column perfusion to conventional treatment was shown to improve the survival in patients with AE-IPF [39, 41]. Rituximab combined with plasma exchange and intravenous immunoglobulin also showed certain benefits [42]. All patients in our study used corticosteroids after AEs, but the maximal dosage was different for each case according to the clinical severity and the extent of damage on chest CT. The overall survival time was not correlated with the maximal dosage of methylprednisolone both in CTD-ILD and IPF patients when AE occurred. So, we speculated that the higher dosage of corticosteroids would not be beneficial for AE patients, and the clinical outcomes of AE patients with pulmonary fibrosis mainly depend on the underlying clinical conditions and the extent of lung injury at AE.

The current study has some limitations. It was designed retrospectively and the stable cases of pulmonary fibrosis were not available as controls. Histological evidence was not available for the diagnosis of pulmonary fibrosis. It was unable to get the exact number of cases with IPF patients. A definite cause of death was not available for each case, although most patients died of AE. A prospective, multicenter, multinational study of a larger cases cohort would be helpful to provide enough data for the epidemiology in AE-CTD-ILD.

## Conclusions

In summary, the current study demonstrated that the clinical characteristics of patients with AE-CTD-ILD differed from those of AE-IPF. The incidence of AE-CTD-ILD was similar to that of AE-IPF. Subjects with AE-CTD-fILD had a better prognosis than those with AE-IPF. However, the survival of AE patients in each group was identical. WBC count and PO_2_/FiO_2_ were the independent predictors for the survival in patients with CTD-fILD. A higher dosage of corticosteroids would not be beneficial for the survival of fILD patien.

## Supplementary information

**Additional file 1: Table S1.** Comparison of the CPS at different time after AE.

## Data Availability

The datasets used and/or analyzed during the current study are available from the corresponding author on reasonable request.

## Abbreviations

AE: Acute exacerbation
ALB: Albumin
BNP: B-type natriuretic peptide
CRP: C reactive protein
CTDs: Connective tissue diseases
DLCO: Diffusing capacity of the lung for CO2
ESR: Erythrocyte sedimentation rate
fILD: Fibrosing interstitial lung disease
FVC: Forced vital capacity
HRCT: High resolution computed tomography
IPF: Idiopathic pulmonary fibrosis
LDH: Lactate dehydrogenase
MV: Mechanical ventilation
NAC: N-acetylcysteine
PAH: Pulmonary arterial hypertension; BMI: body mass index
PaO_2_/FiO_2_: Oxygenation index
P-UIP: Possible UIP
TLC: Total lung capacity
UIP: Usual interstitial pneumonia
WBC: White blood cell
