# Supplementary material for: Acute exacerbations of fibrosing interstitial lung disease associated with connective tissue diseases: a population-based study
Source: BMC Pulm Med. 2019 Nov 14;19:215. doi: 10.1186/s12890-019-0960-1 (PMC6857302; doi:10.1186/s12890-019-0960-1)
Supplement: Supplementary file 1 — Additional file 1: Table S1. Comparison of the CPS at different time after AE. [file 12890_2019_960_MOESM1_ESM.docx]

| **Table S1. Comparison of the CPS at different time after AE.** | | | | |
| --- | --- | --- | --- | --- |
| **Duration after AE (days)** | **AE-IPF group (%)** | **AE-CTD-fILD group (%)** | | **P value** |
| 30 | 53.3 | 62.9 | 0.208 | |
| 60 | 30.8 | 31.4 | 0.934 | |
| 90 | 22.4 | 27.1 | 0.475 | |
| 120 | 14.0 | 25.7 | 0.051 | |
| 180 | 9.3 | 24.3 | 0.007 | |
| 240 | 5.6 | 15.7 | 0.026 | |
